# New Genomic Structure for Prostate Cancer Specific Gene PCA3 within BMCC1: Implications for Prostate Cancer Detection and Progression

**DOI:** 10.1371/journal.pone.0004995

**Published:** 2009-03-25

**Authors:** Raymond A. Clarke, Zhongming Zhao, An-Yuan Guo, Kathrein Roper, Linda Teng, Zhi-Ming Fang, Hema Samaratunga, Martin F. Lavin, Robert A. Gardiner

**Affiliations:** 1 Prostate Cancer Institute, Cancer Care Centre, St George Hospital Clinical School of Medicine, University of New South Wales, Kogarah, New South Wales, Australia; 2 Division of Cancer and Cell Biology, Queensland Institute of Medical Research, Brisbane, Queensland, Australia; 3 Department of Psychiatry and Center for the Study of Biological Complexity, Virginia Commonwealth University, Richmond, Virginia, United States of Amerca; 4 Hopkins Marine Station, Stanford University, Stanford, California, United States of America; 5 Sullivan & Nicolaides Pathology, Brisbane, Australia; 6 University of Queensland Centre for Clinical Research, Brisbane, Australia; East Carolina University, United States of America

## Abstract

**Background:**

The prostate cancer antigen 3 (*PCA3/DD3)* gene is a highly specific biomarker upregulated in prostate cancer (PCa). In order to understand the importance of *PCA3* in PCa we investigated the organization and evolution of the *PCA3* gene locus.

**Methods/Principal Findings:**

We have employed cDNA synthesis, RTPCR and DNA sequencing to identify 4 new transcription start sites, 4 polyadenylation sites and 2 new differentially spliced exons in an extended form of *PCA3*. Primers designed from these novel *PCA3* exons greatly improve RT-PCR based discrimination between PCa, PCa metastases and BPH specimens. Comparative genomic analyses demonstrated that *PCA3* has only recently evolved in an anti-sense orientation within a second gene, *BMCC1/PRUNE2*. BMCC1 has been shown previously to interact with RhoA and RhoC, determinants of cellular transformation and metastasis, respectively. Using RT-PCR we demonstrated that the longer *BMCC1-1* isoform - like *PCA3* – is upregulated in PCa tissues and metastases and in PCa cell lines. Furthermore *PCA3* and *BMCC1-1* levels are responsive to dihydrotestosterone treatment.

**Conclusions/Significance:**

Upregulation of two new PCA3 isoforms in PCa tissues improves discrimination between PCa and BPH. The functional relevance of this specificity is now of particular interest given *PCA3's* overlapping association with a second gene *BMCC1*, a regulator of Rho signalling. Upregulation of *PCA3* and *BMCC1* in PCa has potential for improved diagnosis.

## Introduction

Prostate cancer (PCa) is the most commonly diagnosed internal malignancy in men and the second leading cause of cancer-related deaths. The etiology of PCa is uncertain with environmental, hormonal and hereditary factors implicated. The initiation of PCa (ie. the formation of a histologically identifiable lesion) is a common event, being detected at autopsy series in nearly one-third of men over age 45 [1]. Fortunately the majority of such lesions do not progress to clinically significant tumors. However, in patients with clinically-detected disease and who are considered to have their tumor localized to the prostate, between 15% and 40% have disseminated disease, not identifiable by current imaging methods, for which there is currently no curative treatment. A diagnosis of prostate cancer (from prostatic biopsies) is initiated typically following an elevation in serum measurements of prostate specific antigen (PSA), a protein normally secreted specifically by prostate epithelial cells to form a component of ejaculate. PSA is not a test for cancer and there is no threshold level of this enzyme providing a high sensitivity and specificity with a continuum of risk for all PSA values [2]. A raised serum PSA so often commits men to the invasive and imprecise procedure of transrectal ultrasound (TRUS) guided biopsies [3], [4]. A further indictment of the limitations of PSA in PCa detection is the disparity between TRUS biopsy findings and those from radical prostatectomy with the former under-calling pathology [5].

**Figure 1:**
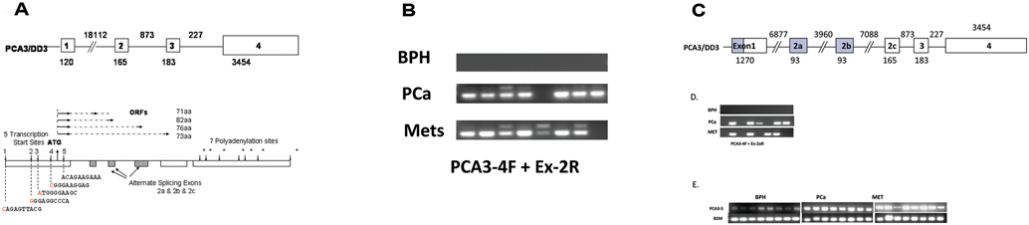
Complexity of *PCA3* transcripts. (A) Partial *PCA3* gene structure as originally reported by Bussemakers et al. [15] with 4 exons (open boxes ∼ not to scale) with alternate splicing of exon 2 and three alternate transcription termination sites in exon 4. 5′ RACE experiments (Supplementary Fig. S1) identified 4 novel *PCA3* transcription initiation sites (isoforms 1–4 marked by vertical arrows pointing down with nucleotide sequence below located 1150 bp, 699 bp, 640 bp and 136 bp upstream of the original initiation site (renamed here isoform 5). 3′ RACE identified four novel polyadenylation sites (7 in total*) located in exon 4. The size of exon 1 is expanded from the original 120 bp to 1270 bp. Isoform 4 (*PCA3-4*) is the most highly expressed of the four novel isoforms. Four overlapping ORFs initiate from a single ‘ATG’ start site (vertical arrow pointing up) within *PCA3-4* and terminate within one of the alternatively spliced exons (2a or 2b or 2c) or within exon 3. (B) RT-PCR amplification of BPH, PCa and PCa metastasis samples using a forward primer from within the novel *PCA3-4* transcription start site and a reverse primer from novel exon 2a. (C) Complete structure of the *PCA3* gene. Shading identifies the newly identified regions of the *PCA3* gene which has 6 exons with alternate splicing of exon 2a (93 bp) and exon 2b (93 bp) and exon 2c (original exon 2, 165 bp). and (D) RT-PCR amplification of PCA3 using the same forward primer and a reverse primer from novel exon 2b and (E) RT-PCR amplification of PCA3 using a forward primer from *PCA3-5F* (within the original transcription start site) and a reverse primer spanning exons 1 and 3 [15].

To improve detection and treatment of PCa, investigations have been on-going to identify the genes involved in the initiation and progression of the disease. Hereditary factors are considered to play a greater role in the genesis of PCa than in any other malignancy. Genomic-wide association studies and candidate gene screens indicate that inheritance involves multiple small associations the vast majority of which remain unknown in addition to possibly complex epigenetic or gene-gene interactions. [6]–[12]. Differential display technology has been used successfully to identify changes in the level of gene expression associated with the transition from normal to tumour which include genes involved in lipid signalling and metabolism; fatty acid synthesis; cell cycle regulation; cell adhesion and stromal regulation; angiogenesis; ion channel regulation, and signal transduction [13], [14]. Using differential display Bussemakers et al [15] identified a cDNA, subsequently named prostate cancer antigen 3 (*PCA3/DD3*), that was upregulated in 53 of 56 prostate cancers when compared with non-malignant prostate tissue. The *PCA3* gene (25 kb – Fig 1A), which is differentially spliced, has a high frequency of termination codons in all reading frames that suggested it was a non-coding RNA (ncRNA). In addition, there was no evidence for expression of a PCA3 protein [16]. Upregulation of the major ∼2 kb *PCA3* transcript, which excludes exon 2 (Fig 1A), was shown to be a sensitive and specific marker for the diagnosis of PCa [15], [16]. The expression of polyadenylated transcripts of *PCA3* suggested that it may have a functional role which is supported to some extent by its localization to the nucleus and failure to be detected in the cytoplasm [16]. To date no role in cancer has been described for *PCA3* but it has been suggested that it may function in regulating gene expression or participate in gene splicing [16]. ncRNAs have been recently found in surprising abundance, with novel classes and unexpected roles mediating evolution, organising chromosomal domains, chromatin remodelling and transcriptional regulation (both activation and suppression) [17], [18]. In addition to *PCA3*, comparisons between benign prostate hypoplasia (BPH) and PCa samples showed 14 of the 51 other ncRNAs that were differentially expressed were also upregulated in PCa [19], [20]. It is also well established that a class of very small ncRNAs known as microRNAs are altered in different tumour types and can act as oncogenes or tumour suppressor genes [19], [21], [22].

In order to understand further the importance of the *PCA3* gene in PCa we undertook a more detailed investigation of this gene and its chromosomal locus. This investigation points to a considerably more complex transcriptional unit for *PCA3* than originally reported [15], [16] including additional novel exons. We describe a number of novel *PCA3* splice variants with more specific expression in PCa tissues and metastases. We also demonstrate that *PCA3* is embedded in the intron of a second gene, *BMCC1*, a gene implicated in controlling oncogenic transformation [23] and that both genes showed increased expression in PCa and metastases.

## Results

### Identification of novel PCA3 transcripts and experience in PCa

The absence of a TATA box element within a human gene promoter has been associated with promiscuous transcriptional initiation. The *PCA3* gene does not contain an upstream TATA sequence and it was therefore of interest to determine whether any additional transcription initiation sites existed for *PCA3* (Fig 1A, upper part). We carried out 5′ RACE using PCa tissue expressing *PCA3* to look for additional start sites.

This approach demonstrated that exon 1 is 1150 bp longer than previously reported (now 1270 bp) and contains 4 novel transcription start sites (Supplementary Fig. S1 & Fig. 1A, lower part). These novel transcription start sites are located 1150, 699, 640 and 136 bp (termed *PCA3* isoforms 1–4, respectively) upstream of the previously reported start site for *PCA3* [15]. The original transcript is referred to here as *PCA3* isoform 5 (*PCA3-5*). The presence of these longer transcripts in tumours was inversely related to transcript length (results not shown). Transcription from novel initiation site 4 (*PCA3-4*), juxtaposed immediately downstream of the FP2 region containing the 3 *SRY* consensus binding sites (Supplementary Fig. S2), was significantly higher compared with the other three upstream initiation sites as judged by qPCR (results not shown). Transcription from initiation site 1 (*PCA3-1*) was detected by 5′ RACE in only a few samples.

We carried out 3′ RACE to investigate any further complexity at the 3′ end of the transcript. Four additional polyadenylation sites were detected using 3′ RACE bringing the total number of polyadenylation sites to seven, located at nucleotides 411, 542, 873, 1583, 1600, 2146 and 3545 respectively in exon 4 (Fig. 1A, lower part). Of the 4 additional polyadenylation sites, only two were associated with defined polyadenylation signal sequences, AATAAA and ATTAAA respectively. We observed that a forward primer based on the new sequence (*PCA3-4)* together with a reverse primer for exon 2 very efficiently amplified *PCA3* in PCa (7/8) and metastases samples (7/8) but failed to detect *PCA3* in BPH samples (0/8) (Fig 1B). Clinical information on these patients is provided in Supplementary Table S1. These primers not only amplified a cDNA fragment of the expected size (265 bp) but also 2 higher molecular size bands in some samples (Fig 1B, Supplementary Fig. S3). The additional bands were excised from the gel and sequenced to reveal the presence of 2 novel *PCA3* exons both 93 bp in size (Fig 1C). These two differentially spliced exons (2a and 2b) which have bona-fide consensus splice sites at their ends had their expression confirmed by RT-PCR amplification using novel exon specific primers Amplification of *PCA3* using the PCA3-4F primer together with a primer corresponding to exon 2a detected 5/8 PCa and 4/8 metastatic samples and again provided excellent discrimination with BPH (Fig 1D). Similar results were demonstrated using PCA3-4F and a reverse primer for exon 2b (results not shown). This improves on the use of the original primer set employed by Bussenmakers et al [15] which also detected a less intense signal in most BPH cases as is evident from data obtained here (Fig. 1E). The identification of exons 2a and 2b brings to 6 the total number of *PCA3* exons (Fig. 1C). These data indicate that multiple novel transcripts for *PCA3* are differentially expressed in PCa.

Nucleotide sequence analysis identified four putative ORFs initiating from a single ATG located 54 nucleotides within the novel *PCA3-4* isoform (Fig 1A, lower part). The first of these, 70 amino acids (aa) in length, extended through exons 1 and 4 aa into a novel exon 2a. The second, 82 aa in length, extended through exon 1, skipped exon 2a, and extended 16 aa into exon 2b. The third, 76 aa in length, also extended through exon 1, skipped exons 2a and 2b and extended 10 aa into exon 2c. The fourth, 73 aa in length, also extended through exon 1, skipped exons 2a and 2b and 2c and extended 7 aa into exon 3. These ORFs initiate 83 nucleotides upstream of the original transcript (*PCA3-5*) described by Bussemakers et al. [15] and could not have been predicted in previous analyses of the original transcript which did not identify any significant ORFs. It will be of interest to determine whether these ORFs code for proteins.

### *PCA3* is embedded within intron 6 of the *BMCC1* gene

**Figure 2:**
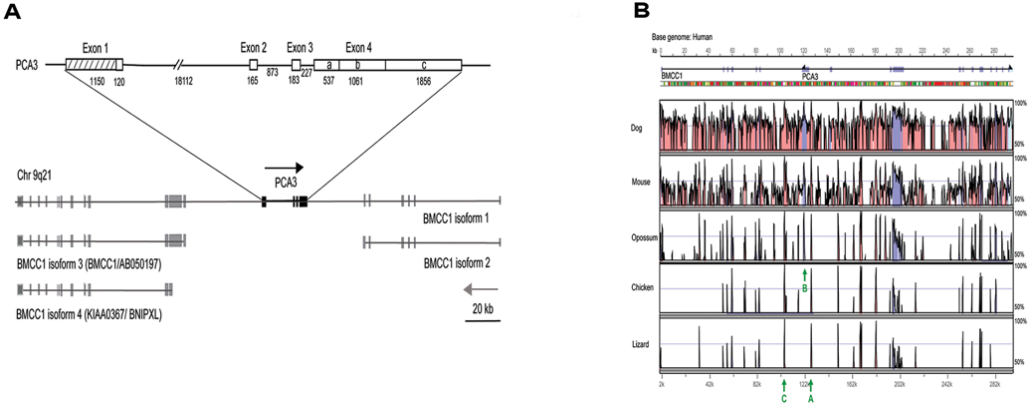
*PCA3* is embedded within *BMCC1*. (A) The *PCA3* gene (above) is embedded within the intron 6 of *BMCC1* (*PRUNE2*) isoform 1 (*BMCC1-1*). Gray boxes denote exons of *BMCC1* and black boxes denote exons of *PCA3.* The two genes are in the opposite orientation (NCBI Build 36). Three other isoforms of *BMCC1* have also been described, none of which include the complete set of exons present in *BMCC1-1*. (B) VISTA plot of *BMCC1* gene. Peak heights indicate degree of conservation between species of exons (blue) and evolutionary conserved regions (ECR within introns - pink) compared with human. Note that gene orientation is different in Fig 2A, upper and lower panels. We estimated the mutation rate at the DNA sequence level by comparing human and chimpanzee sequences and using a divergence time of 6 million years (Myr). The mutation rate in the *PCA3* gene was estimated to be 1.26×10^−9^ per nucleotide per year, higher than that (1.00×10^−9^ per nucleotide per year) in the non-*PCA3* portion of the *BMCC1* gene, suggesting the *PCA3* region might have a moderately higher mutation rate. Three highly conserved ECRs within *BMCC1* are arrowed (A, B & C); Arrow ‘A’ (ECR_in1, 277 bp) is an extremely conserved non-coding sequence with 91% similarity between human and opossum that is positioned within intron 1 of *PCA3*; Arrow ‘B’ (ECR_ex4c, 403 bp) an ECR positioned within *PCA3* exon 4 which is conserved in all mammals and appears to have been the focal point for the linear evolution of the *PCA3* gene (see Fig. 3); Arrow ‘C’ (361 bp) is the most highly conserved ECR (a conserved non-coding sequence with 99% similarity between human and opossum) within *BMCC1* (intron 6) immediately downstream of *PCA3*.

**Figure 3:**
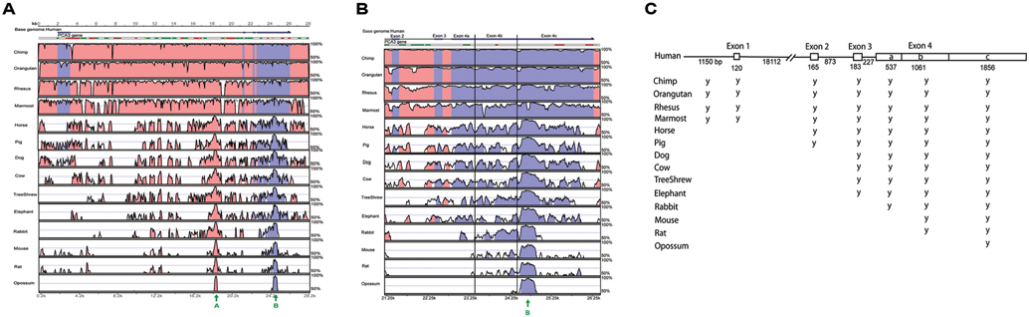
Linear evolving structure (3′→5′) of the *PCA3* gene. (A) Vista plot displaying conserved structures of the *PCA3 gene*. Only primates appear to have a complete *PCA3* gene. The two evolutionary conserved regions shared by *BMCC1* and *PCA3* (see Fig. 2B) *are arrowed* (arrow A ∼ ECR_ex4c and arrow B ∼ ECR_in1). Both ECRs appear conserved in mammals (identity >90%). ECR_in1 is also present at this site in chicken and lizard but not in fish, frog, or invertebrates. (B) ECR_ex4c appears focal to the linear evolving structure of *PCA3*. (C) Summary of mammalian *PCA3* exons sharing high conservation compared to human. The gene structure annotation was based on Bussemakers et al. [15]. The level of conservation of *PCA3* exons during the course of mammalian evolution increases 3′→5′ based on the presence of meaningful ECRs in each exon. The exact sequence identity for the complete exon between human and other species is shown in Supplementary Table S2. It is important to note that no ECR from *PCA3* exons was found in any non-mammalian species in this analysis.

To understand better the close correlation between *PCA3* gene expression levels and prostate cancer we investigated the evolution and organisation of the *PCA3* gene locus (Fig 2A). We used the *PCA3* mRNA sequence (3923 bp, AF103907) to search homologous sequences in other genomes. Using a cutoff E-value <1×10^−4^ in the BLAST search, significant hits were found only in mammalian genomes. Exon 4 is the most conserved region of the gene. One segment (403 bp) in exon 4 was found in all the available mammalian genomes (evolutionary conserved region- (ECR_ex4c arrowed ‘B’ in Figs 2B and 3A & 3B). This segment, along with the human *PCA3* gene sequence, was used to extract the genomic sequences for *PCA3* homologs in other species. As a result, we obtained human *PCA3* homologs in 14 mammals, including 4 non-human primates.

**Figure 4:**
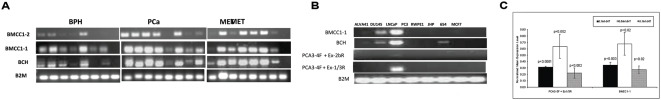
*PCA3* and *BMCC1-1* expression patterns in prostate cancer. cDNA was prepared from patient tissue specimens including eight BPH, PCa or PCa metastases, respectively, for use in the following PCR reactions. (A). RT-PCR carried out on BPH, PCa and metastases (MET) with different sets of primers for *PCA3* and BMCC1 Upper row; *BMCC1-2* RT-PCR using a forward primer for *BMCC1* exon 5 (BMCC1-Ex5F) and a reverse primer specific for the extended form of exon 6 (BMCC1-Ex7R) unique to *BMCC1-2* 2nd row; *BMCC1-1* specific RT-PCR using primers for *BMCC1* exon 6 (BMCC1-Ex6F) and exon 7 (BMCC1-Ex7R). 3rd row; *BMCC1*-BCH region specific RT-PCR using primers BCHF and BCHR. 4th row; *PCA3* was amplified (35 cycles) with primers specific to *PCA3* isoform 5 exon 1 (PCA3-5F and the Ex1/3R primer). 5^th^ row; β_2_microglobulin control PCR (B). RT-PCR comparative expression analysis of *PCA3*, *BMCC1-1* and the *BMCC1*-BCH region for ALVA41, DU145, LNCaP and PC3 prostate cancer lines, RPWE1 a normal prostate cell line, JHP a control lymphoblastoid cell line, RM654 a lymphoblastoid cell line from a patient with AOA2 and MCF7 a breast cancer cell line. RT-PCR was carried out with the primer sets specific for *PCA3* isoform 5 (PCA3-5F) and Exon 1/3 (Ex1/3R) and for *BMCC1-1* and the *BMCC1*-BCH region as indicated above. (C) Semi-quantitative PCR analysis of *PCA3* and *BMCC1-1* expression in the LNCaP cell line in response to dihydrotestosterone. Results were normalised relative to levels of β_2_microglobulin. Four cell cultures were starved of serum for 2 days prior to incubation with dihydrotestosterone (µg/ml). Results were normalised and expressed as mean fold increase relative to the level of expression before treatment. Error bars are standard deviations and p values were determined from comparison with untreated samples using a Student's t-test.

Two mRNA sequences (AB050197 and BC019095) were initially annotated upstream and downstream of *PCA3*. These two mRNA sequences were recently merged and annotated as two isoforms of the *BMCC1* gene (also called *PRUNE2*, NCBI Gene ID: 158471). According to these mapping locations, the *PCA3* gene is located within intron 6 of the longer *BMCC1* isoform 1 (*BMCC1-1*). To confirm this we searched for *PCA3* sequences elsewhere in the human genome and obtained only one hit which exactly maps to the *BMCC1* gene locus. The *BMCC1* gene is ∼295 kb in length and has an opposite gene orientation to *PCA3*. Recent expression studies indicate that *BMCC1* processing is more complex than initially thought and comprises four variant isoforms that were not fully annotated on NCBI (Build 36.2). Fig. 2A illustrates the structural relationship between the *PCA3* and *BMCC1* genes. *BMCC1* isoform-2 (*BMCC1-2/PRUNE2-2*/BC019095/NM_138818) comprises the first 6 exons of *BMCC1* as annotated on NCBI (Build 36.2). *BMCC1-2* terminates immediately upstream of the *PCA3* gene. Isoform-3 (*BMCC1-3/BMCC1*/ABO50197) reported by Machida et al. [23] does not overlap *BMCC1-2* but rather comprises 13 distinct exons (exons 7–19) positioned immediately downstream of *PCA3*. The transcription initiation site for isoform-4 (*BMCC1-4*/KIAA0367/BNIPXL/AY43213) reported by Soh and Low [24] is located still further downstream within the second exon of *BMCC1-3*. Isoform-1 (*BMCC1-1/PRUNE2-1*/NM_015225) on the other hand, is a recent computationally generated reference gene assembly comprising the 19 exons (NM_015225) derived from merging *BMCC1-2* and *BMCC1-3* and which had, until now, lacked full transcript support. Here we used RT-PCR primers spanning *BMCC1-2* and *BMCC1-3* (Fig. 2A) and nucleotide sequence analysis (results not shown) to verify the existence of the *BMCC1-1* transcript and confirmed its expression in PCa tissues (See Fig. 4A). The larger size of *BMCC1-1* is also consistent with the ∼12 kb mRNA transcript identified by Machida et al. [23]. *PCA3* locates within intron 6 (∼110 kb) of *BMCC1-1*. *PCA3* represents approximately 25 kb of this intron but it is in the opposite orientation to *BMCC1*. Three of the BMCC1 protein isoforms BMCC1-1 (3088 aa – NM_015225), BMCC1-3 (2724 aa) [23] and BMCC1-4 (769 aa) [24] have different coding start sites, however, all three are in-frame and contain the downstream BCH coding domain.

### Has the BMCC1 gene been under selection?

Since *PCA3* is embedded or nested within *BMCC1* it was of interest to study evolutionary changes in this gene. BLAST searches revealed that *BMCC1* homologs are present in mammals, chicken and lizard but not in African frog, fish or invertebrates (Fig 2B). We used VISTA software to perform global alignment of *BMCC1* homolog genes. The alignment in Fig 2B demonstrates that *BMCC1* is only conserved from human to dog/mouse with the exception of a number of extremely conserved ECR that extend from human to lizard (including those arrowed A and C in Fig. 2B).

These ECR are located largely between *BMCC1* intron 6 and exon 9 (including within *PCA3*). We compared amino acid sequences based on human and chimpanzee *BMCC1-1* CDS sequences. The total amino acid length is 3088 (human) and 3089 (chimpanzee). After we aligned human and chimpanzee sequences, we found 40 non-synonymous mutations and 21 synonymous mutations. For the whole region, the ratio of non-synonymous over synonymous substitution rate (dN/dS) is 0.90. A dN/dS ratio greater than 1 in a coding region often suggests positive selection. Exon 8 is the longest exon in *BMCC1* and has 6598 bp, which encodes 2199 amino acids. We found the majority of non-synonymous mutations are in exon 8 and the number of non-synonymous mutations [25] is almost three times that of synonymous mutations [12]. The dN/dS ratio was 1.38, suggesting a recent positive selection at this subregion. Furthermore, we found that 39 of the 40 non-synonymous substitutions in the *BMCC1* gene were in exons 8 and 9, which had only 14 synonymous substitutions. There was only one substitution, which is synonymous, in exon 7. When we examined exons 8–9 or exons 7–9, we consistently found that the dN/dS ratio was greater than 1 (exons 8–9, 1.30; exons 7–9, 1.22), suggesting a positive selection in the neighbouring region of *PCA3*.

### *PCA3* emerged in mammals and recently evolved in primates

*PCA3* is not as well conserved in mammals as *BMCC1* and was not detected previously in rodents [15]. To determine the origin of *PCA3* we compared *PCA3* gene sequences across species. We performed a global alignment of the 15 mammalian *PCA3* orthologs. Fig. 3A displays a VISTA plot of the conserved regions within the *PCA3* gene region. A more detailed VISTA plot focusing on *PCA3* exons 2–4 is provided in Fig. 3B. As described, the *PCA3* gene is highly conserved in primates. For example, when compared with the human *PCA3* sequence, all four exon sequences and the majority of the intron sequences in the four primates have high identity with human (Fig. 3A).

Comparison of sequence conservation among these 15 mammals, revealed a linear pattern of change relative to these four exons during the course of mammalian evolution. Exon 4 is by far the largest exon (3454 bp) and for comparative purposes exon 4 was divided (5′→3′) into 3 regions [a, b and c ~ corresponding to the three termination sites described by Bussemakers et al. [15]. ECR_ex4c, the conserved segment of exon 4c described earlier (arrowed ‘B’ in Fig. 2B and Fig. 3) appears in all 15 mammals including the opossum, which is an out-group of the 14 eutherians. While opossum has only this one conserved region relative to the 4 *PCA3* exons, the rodents have 1–2 small additional ECRs in exon 4b (Fig. 3A and 3B). Exon 4a appeared first in rabbit. Exon 3 was detected in elephant, tree shrew, cow, dog, pig, horse, and in all primates, but not in rabbit, rodents, or opossum. According to the VISTA plot a meaningful exon 2 is only present in pig, horse and all primates (Fig. 3B).

Finally, exon 1 is present in primates only (Fig. 3A). This linear evolutionary pattern of gene formation is summarized in Fig. 3C where results suggest that: (1) vestiges of the *PCA3* gene emerged in mammals; (2) these vestiges subsequently underwent a linear pattern of evolution: exon 4c→exon 4c/4b→exon 4c/4b/4a→exons 4/3→exons 4/3/2→exons 4/3/2/1; and (3) *PCA3* appears to mature in primates with all sharing a full complement of exons (Fig. 3C). The exact sequence identity for each of the complete human *PCA3* exons compared with other species is shown in Supplementary Table S2.

### BMCC1 is upregulated in PCa and androgen inducible

Since *PCA3* is upregulated in PCa and since we showed here that this gene is embedded in a second gene *BMCC1*, implicated in cellular proliferation, we determined whether *BMCC1* was also differentially regulated in PCa. We used a set of RT-PCR primers that span that region of the *BMCC1* gene (exons 6 and 7), specific for the full-length *BMCC1-1* transcript. Expression of *BMCC1-1* was evident in normal prostate and BPH specimens and was upregulated in PCa and metastases (Fig 4A, Supplementary Fig. S4). This was confirmed using primers corresponding to the BCH C-terminal region of BMCC1 and for BMCC1-2. Indeed amplification of this isoform gave better discrimination between PCa and BPH (Fig. 4A, upper panel). Extending these experiments to PCa and other cell lines revealed that both genes were highly expressed, specifically in the PCa cell line LNCaP (Fig. 4B). In addition *BMCC1-1* was detected in a second PCa cell line DU145 but at lower levels. *PCA3* is also expressed in DU145 but required further rounds of amplification for detection. The shorter *BMCC1* isoforms (*BMCC1-3* and/or *BMCC1-4*) were also detected (using primers specific for the BCH region) in an EBV-transformed lymphoblastoid cell line (JHP), but the longer *BMCC1-1* isoform was not detected (Fig 4B). Previous data have shown that the level of *PCA3* can be induced in LNCaP cells after treatment with dihydrotestosterone, which mimics the effects of binding of the androgen receptor (DHT) [16]. We determined whether *BMCC1-1* was also responsive to hormonal induction. The results in Fig. 4C demonstrate that both *PCA3* and *BMCC1* are maximally induced in the LNCaP cell line at a concentration of 0.5 mM DHT.

## Discussion

We have revealed here a number of novel findings for the *PCA3* biomarker gene that is dramatically upregulated in PCa. Bussemakers et al. [15] had previously shown that *PCA3* consisted of 4 exons and that different transcripts arose due to alternate splicing of exon 2 and the presence of 3 polyadenylation sites in exon 4. Our data reveal that the transcriptional unit for *PCA3* is considerably more complex than this. In addition to the transcription start site reported by Bussemakers et al. [15] we have identified 4 additional transcription start sites extending upstream by over 1 kb which increases the size of exon 1 to 1.27 kb. The transcripts initiating at these novel sites are differentially expressed with the shorter isoform 4 (*PCA3-4*) more highly expressed in PCa and metastases. Schalken et al. [16] established that a fragment of 500 bp immediately upstream from the original transcription start site (described here as *PCA3-5*) has all the critical activator and repressor sites to drive *PCA3* expression. Our description of additional *PCA3* start sites further upstream of the two shorter isoforms (*PCA3-4* and *PCA3-5*) is contained within a larger transcriptome and that it is likely that other control elements exist further upstream. This arrangement of the transcriptome is not novel as many genes are arranged in complex overlapping and interlaced patterns in eukaryotic genomes [26]. In that report bypassing mechanisms are invoked for processing at the 3′ end of the transcript. This may also be the case at the 5′ end. We also describe 2 new differentially spliced exons (exons 2a and 2b) for *PCA3* which are located between exon 1 and the original exon 2 (now exon 2c) [15]. Transcripts containing sequence from any or all of these alternatively spliced exons are expressed at low levels and were detected primarily in PCa specimens and metastases. RT-PCR specific for these novel, alternatively spliced exons provides real potential to improve discrimination between BHP and PCa and metastases.

Another novel aspect of this study is the demonstration that the *PCA3* gene is embedded or nested within intron 6 of the *BMCC1-1* gene. Our results indicate that this overlap between the two genes appears to have recently evolved through concurrent evolutionary changes to both *BMCC1* and *PCA3* genes. Both *PCA3* and the full length *BMCC1* isoform, *BMCC1-1*, appear to have recently evolved. Only fragments of the *PCA3* gene are evident in the non-primate mammals tested and exons 7–9 of *BMCC1-1*, immediately upstream of the *PCA3* gene, are also poorly conserved in non-primate mammals. This is consistent with hybridization data by Bussemakers et al. [15] showing the presence of *PCA3* gene sequences in monkey, cow, pig, goat and sheep, faintly detected in the dog but absent in rodents. The detection of vestiges of the *PCA3* gene within the *BMCC1* gene in non-primates suggests that *PCA3* has evolved in concert with a central section of *BMCC1* (between exons 7 and 9). This contrasts with the relative conservation of *BMCC1* exons 1–6 (corresponding to the *BMCC1-2* isoform) and exons 13–16 which span the BCH coding region shared by BMCC1-1 with BMCC1-3 and BMCC1-4 [23]. *PCA3* is transcribed in an anti-sense orientation relative to *BMCC1* which could lead to interference with BMCC1 expression. However, the fact that both *PCA3* and *BMCC1-1* are both upregulated in PCa and PCa metastases could indicate a positive level of coordinated control and explain their paralleled evolutionary selection at least in humans.

This is the first report to describe the longer *BMCC1* isoform 1 (*BMCC1-1*) and the first to demonstrate *BMCC1-1* expression patterns. We provide evidence for the expression and upregulation of *BMCC1-1* and other *BMCC1* isoforms in PCa and metastases. In a previous report which evaluated expression of BMCC1-4 (BNIPXL) the BCH domain at the C-terminus, which is homologous to the BCH region of the *BNIP2* and *BPGAP1* genes, was shown to target Rho proteins with potential to inhibit cellular proliferation [24]. This inhibitory effect, however, was moderated with the inclusion of an increased N-terminal sequence from BMCC1-4 (BNIPXL, 769 aa), an arrangement that may be further moderated or altered with the inclusion of the vastly increased N-terminal sequence from BMCC1-1 (3088 aa), which has yet to be tested. Specifically, the BCH domain of BMCC1 targets RhoA and RhoC (members of the Ras superfamily of small GTPases that cycle between inactive GDP-bound and active GTP-bound states) and Lbc and p115RRhoGEF (RhoA-specific guanine nucleotide exchange factors). Both RhoA and RhoC induce stress fibers. RhoA participates in oncogenic transformation whereas RhoC promotes tumor metastasis and cell migration [27], [28]. Rho proteins also regulate cell morphology, motility, vesicular transport, membrane trafficking, lipid signalling, cell cycle progression and gene transcription and dysfunctional regulation of Rho signalling leads to cancer [27], [29]. Overexpression of the BCH domain reduces active RhoA levels while knockdown has the reverse effect [24]. *BMCC1* upregulation (evaluated using downstream RT-PCR primers that span exons 8 and 9) in human neuroblastoma correlates with a more favourable prognosis consistent with a role in inducing apoptosis [23]. In this study we observed upregulation of *BMCC1-1*, *BMCC1-2* and the BCH region shared by *BMCC1-1* with *BMCC1*-3 and *BMCC1-4*, in both PCa and metastases. However, it is not yet clear what role the combined upregulation of the various BMCC1 isoforms may play in PCa.

We have also shown here that *BMCC1-1* expression is responsive to androgen treatment. The coordinated control of these two overlapping genes could operate through the action of an androgen responsive transcription factor like SRY [25] and/or through chromatin affects and/or the effects of trans-acting enhancer elements that may include the extremely conserved non-coding sequences identified here within and adjacent to the *PCA3* gene (ECRs arrowed B&C in Fig. 2B) [27]. SRY is of particular interest since it interacts with and negatively regulates androgen receptor (*AR*) activity [25]. AR appears to suppress epithelial proliferation in the mature prostate; therefore, any significant increases in SRY could be linked to increased epithelial proliferation [25]. It is possible that the co-expression of *PCA3* and *BMCC1* has an activation effect. It is well established that enhancers play an important role in chromatin opening to facilitate transcription activation and immunoglobulin gene recombination [30]. In the present case enhanced transcription of *PCA3* may open up the locus and in turn increase the level of *BMCC1* transcription. The shortest BMCC1 isoform 4 (BMCC1-4/BNIPXL) may have pro-apoptotic affects [24] but this has not been tested in PCa and the role of the much longer BMCC1 isoform 1 (BMCC1-1) has yet to be investigated. The BCH domain at the C-terminus of BMCC1 facilitates homodimerisation and heterodimerisation with other proteins containing a BCH domain [23], [24]. The significance of these homo and heterodimeric interactions, particularly as they relate to Rho signalling, must now be re-evaluated because of the existence of multiple BMCC1 isoforms and their upregulation in PCa and metastases.

While we have demonstrated that *PCA3* and *BMCC1-1* are both upregulated in PCa it is important to point out that they are transcribed in the opposite orientation and thus it is possible that the *PCA3* transcript or regulatory factors involved in *PCA3* transcription or its suppression or splicing/processing could directly influence the transcription or processing of the primary *BMCC1-1* transcript during cancer development. *PCA3* is a putative ncRNA and ncRNAs are known to play important roles in transcriptional regulation (both activation and suppression), gene silencing, RNA splicing, and DNA imprinting and demethylation [17]. ncRNAs are involved in many diseases including cancer and neurological disorders [18]–[22], [31]. *PCGEM1* is another example of a ncRNA found over-expressed in prostate cancer [20] but it differs from *PCA3* in that it does not locate in an intronic region of another gene. Our finding that *PCA3* locates within an intron of the *BMCC1* gene and is transcribed in the opposite orientation suggests that it may serve as an intronic anti-sense transcript. Intronic anti-sense transcripts may play important roles in PCa; for example, Reis et al. [32] found that 6 of the top 12 transcripts that were most correlated to prostate tumor differentiation were intronic anti-sense transcripts. In the mouse genome, Kiyosawa et al. [33] identified 899 pairs of transcripts, in which one transcript lies in an intron(s) of another transcript and has an opposite orientation. Overlapping genes are a relatively common feature of eukaryotic genomes where, like *PCA3*, they are often found embedded/nested entirely within an intron of the other gene [33], [34]. For example, intron 27 of the human neurofibromatosis type 1 gene has three embedded genes: *OMG*, *EV12B*, and *EV12A*. Most overlapping genes are transcribed in opposite orientations and generate natural anti-sense transcripts [33], [35] and a growing body of evidence indicates the potential for overlap to affect gene regulation. For example, an overlap between the *rTSalpha* and *thymidylate synthase* (*TS*) genes causes site-specific cleavage and down-regulation of the *TS* mRNA through a natural RNA-based anti-sense mechanism [36]. Another example comes from the two overlapping isoforms of the human *c-erbAlpha* gene where natural anti-sense transcripts inhibit the alternate splicing of mRNA, probably by blocking the accessibility of *cis* regulatory elements [37]. It has been proposed that *SRY* also has potential to act as a direct modulator of RNA splicing [38]. The multiple isoforms and alternate splicing of both *PCA3* and *BMCC1* provide avenues for similar investigations.

In summary we have demonstrated greater complexity in *PCA3* transcripts than previously reported and shown that the complete *PCA3* gene is embedded in the intron of a second gene, *BMCC1*. Both of these genes are upregulated in PCa and are androgen responsive. At this stage it is not clear whether *PCA3* or associated regulatory factors interfere with or enhance the expression and function of BMCC1 proteins. However, it is intriguing to speculate that this may be the case and it will be of great interest to investigate how this might impact on the development of PCa. The differential splicing and expression of 2 novel exons, exon 2a and 2b, which are highly enriched in PCa and metastases promises to add a further degree of sensitivity for the detection of PCa. At present the use of RT-PCR to detect expression of *PCA3* in post-prostatic, massage urine is available commercially as a test for prostate cancer (www.PCA3.org). The preliminary data described here using additional sequence information on the *PCA3* gene together with the observation that another gene BMCC1 is also overexpressed in PCa, increase the potential to provide a better diagnostic test as well as a prognostic tool in predicting tumour development or aggressiveness.

## Materials and Methods

### Specimen collection

All tissue specimens were collected following written consent at Royal Brisbane and Women's Hospital, Queensland, as approved by the Royal Brisbane and Womens Hospital Ethics Committee. Primary prostate cancer tissue specimens were obtained from patients undergoing radical prostatectomy or transurethral resection of prostate with secondary tumours harvested from lymph node metastases in patients with castrate-resistant prostate cancer. Tissue was obtained from the radical prostatectomy specimens by open biopsy from the region identified by TRUS biopsy findings. To ensure that there was a high probability of cancer being present, sections were cut from all four sides of the specimen and which confirmed malignancy. The presence of cancer in the TURP fragments was confirmed by the same process. The 3 patients who had radical prostatectomies (PCa1, PCa3, PCa8 – Supplementary Table S1) had clinically localized prostate cancer (margin negative and seminal vesicle negative) but with 30–60% of the glands containing tumour). PCa2 and PCa6 had castrate – resistant prostate cancer having received bilateral orchidectomy and LHRH against therapy, respectively. PCa4 and PCa7 had metastic prostate cancer on presentation. PCa4 commenced androgen deprivation therapy during his recuperation immediately following TURP and PCa7 had a bilateral orchidectomy immediately following TURP under the same anaesthetic. PCa5 had a T2c stage tumour. Mets 1–8 had lymph nodes harvested by open and laproscopic procedures to provide antigen for a vaccine study. All had castrate – resistant prostate cancer with metastatic bone disease evident on radioisotope bone scan for patients 1, 5 and 7.

Benign prostatic hyperplasia (BPH) tissue specimens were obtained from men who underwent either transurethral resection of the prostate (TURP) or an open enucleative prostatectomy. Tissue fragments were frozen immediately using liquid nitrogen and transported on dry ice for storage at −70°C with closely adjacent tissue specimens placed in OCT and snap frozen or formalin fixed and paraffin-embedded. Tissues prepared for histology immediately adjacent to harvested specimens (BPH and PCa 1-8, Supplementary Table S1) were examined. In adition confirming the diagnosis of BPH or prostate cancer, respectively sections were also examined to determine the proportion of epithelial cells to stromal cells.

### RNA isolation and cDNA synthesis

Total RNA was extracted from prostate tissues using Trizol (Invitrogen) following manufacture's protocol. Subsequent DNase treatment was performed with DNase I (NEB Biolabs: Cat No. M0303S), ethanol precipitated, resuspended in DEPC-treated water and quality controlled via spectrophotometry and gel electrophoresis. All RNA was confirmed to be of good quality and thus suitable for subsequent experiments if the A260/280 ratio was >1.7 and little RNA degradation was evident by gel electrophoresis. 1 µg of total RNA extracted was reverse transcribed using 250 ng of random hexamers (Promega) in a standard 20 µl reaction including 4µl of first strand buffer (Invitrogen), 2 µl of 0.1M DDT (Invitrogen), 1 µl of 10 mM dNTP (Promega), 1 µl RNase inhibitor (2500 U) (Promega) and 1 µl of reverse transcriptase (10,000 U) (Invitrogen). After annealing of the hexanucleotides for 10 minutes at 72°C, cDNA synthesis was performed for 42°C for 90 minutes followed by an enzyme inactivation step at 70°C for 15 minutes. All cDNA products were diluted in a ratio of 1:10 and stored at −20°C before use.

### Search for PCA3 gene in different species

To search the *PCA3* homologous genes in non-human genomes, we performed BLASTN search of the longest *PCA3* mRNA sequence (accession ID: AF103907) against all the publicly released genomes deposited in the Ensembl (http://www.ensembl.org/) or the NCBI (http://www.ncbi.nlm.nih.gov/) databases. We also performed BLASTN search against several genomes (orangutan, marmost, rabbit, elephant and tree shrew) that have been completed but not officially published. These genome sequences were retrieved from the genome sequencing centers at the Washington University in St. Louis (http://genome.wustl.edu/) and the Broad Institute (http://www.broad.mit.edu/mammals/).

### Evolutionary conserved regions (ECRs)

ECRs were identified by VISTA (http://genome.lbl.gov/vista/) [39] with the human sequence as the reference. An ECR was defined as an alignment with a minimum length of 100 bp and at least 70% identity [40]. We analyzed ECRs by sequence comparison of human with other species.

### Molecular evolutionary analysis

Alignment of multiple genomic sequences was performed by ClustalW (v1.8.3) [38]. Detection of signature of adaptive selection was performed by PAML (version 4) [40]. Specifically we used the yn00 program in the PAML package to calculate the ratio of non-synonymous over synonymous substitution rates (dN/dS) between human and chimpanzee *BMCC1* genes. We estimated the mutation rate in the *PCA3* gene and non-*PCA3* portion of the *BMCC1* gene. The mutation rate was estimated by the nucleotide substitution rate between human and chimpanzee sequences using a human–chimpanzee divergence time 6 million years ago [41].

### Identification of transcription start sites: 5′RACE

1 µg of total prostate tissue RNA were reversed transcribed using 1 µl of reverse transcriptase (10,000 U) (Invitrogen), where each reaction was primed with 1 µl of 12 µM 5′-CDS primer A (5′-(T)_25_VN-3′) (Clontech) and 1 µl of 12 µM SMART II A oligo (5′-AAGCAGTGGTATCAACGCAGAGTACGCGGG-3′) (Clontech). After annealing of the hexanucleotides for 10 minutes at 70°C, cDNA synthesis was performed using Superscript II (Invitrogen) at 42°C for 90 minutes followed by an enzyme inactivation step of 72°C for 7 minutes with addition of 100 µl of Tricine-EDTA buffer. The 5′RACE clones were amplified with 5′ RACE DD3 primers (5′GCAGGTGGCCACTCCCATCATGCAAG - 3′) and 10× Universal Primer A mix (Clontech) following manufacturer's protocol (Clontech). The following PCR program was applied: 5 cycles of 94°C for 30 seconds and 72°C for 2 minutes, 5 cycles of 94°C for 30 seconds, 70°C for 30 seconds and 72°C for 2 minutes, and 30 cycles of 94°C for 30 seconds, 68°C for 30 seconds and 72°C for 2 minutes. The 5′RACE PCR products generated were excised and clone into pGEMT (Promega) vector. Positive clones generated were sequenced using Big Dye chemistries.

### 3′ rapid amplification of cDNA ends (3′-RACE) of PCA3 transcripts

3′-RACE libraries were generated from normal, BPH, PCa, and metastatic PCa RNA with Superscript III (Invitrogen: Cat No. 18080-093) using primers and protocols described in the SMART RACE User Manual (BD Biosciences Clontech). Subsequent PCR was performed using a gene specific primer located in exon 3 (5′-CCACACACACAGGAAGCACAAAAGG-3′). As the largest product from this PCR was only 1.5 kb and previous reports described a much longer expected transcript [1], [2], a second primer (5′-GGGCACTCTTGTGAGCCACTTTAGGG-3′), located in exon 4, was designed to “walk” along the *PCA3* transcript. To ensure no further or longer transcripts were present, a third primer (5′-CCCACCACTAACCTGAATGCCTAGACCC-3′) was designed at the end of the last transcript identified using the second primer, which corresponded to the area just upstream of the previously reported end of exon 4 [15], [16]. This RACE PCR resulted in no products as visible by gel electrophoresis, indicating exon 4 does not extend further then previously reported.

### Non-quantitative RT-PCR

Primers for cDNA-specific RT-PCR assay were designed as described below:

1. Primers for *PCA3*
2.
  - PCA3-5F, 5′- AGAAATAGCAAGTGCCGAGAA-3′,
  - Ex2R, 5′- ACTCAGAAAGTGCCGTCGAT-3′
  - PCA3-4F, 5′- TATTCTGAAGTCAGAGTGTTCCAG - 3′
  - Ex1/3R, 5′ - CTTATTTCTCACCTCTGTATCATCAGG - 3′
  - Ex2aR, 5′- GTACCTGCCTTCATGTCACATTG - 3′.
3. Primers for *BMCC1*
4.
  - BMCC1-Ex5F, 5′-TTTCAAGTGGATGACCATGGAATCAG -3′
  - BMCC1-Ex6R, 5′ -CAGACTGCAATTGTGGGAAATCAATC -3′
  - BMCC1-Ex6F, 5′-CTAAAGGAGCTGTCAGATGG -3′
  - BMCC1-Ex7R, 5′ -GAGTACACAGCAATCTGTCG -3′
  - BCHF, 5′ – ATCATTGTGTTTGCCGCCTG -3′
  - BCHR, 5′ – CTTCTTCCAGCATGGCCAACTAAGGC -3′
5. Other Primers
6.
  - SRYF, 5′ - TCCTCAAAAGAAACCGTGCAT -3′
  - SRYR, 5′ – AGATTAATGGTTGCTAAGGACTGGAT -3′
  - PSAF, 5′ – GCATCAGGAACAAAAGCGTG – 3′
  - PSAR, 5′ – CCTGAGGAATCGATTCTTCA -3′
  - β_2_MF, 5′ – GTCTTTCTATCTCTTGTACTACACTGAA -3′
  - β_2_MR, 5′ – AACTATCTTGGGCTGTGACAAAG -3′

Non-quantitative RT-PCR was carried out on a PCR thermocycler (MJ research) with gene specific primers. Each reaction contained 5 µl of the diluted cDNA template, 2.5 µl of 10× PCR buffer, 0.2 µl of 25 mM dNTPs, 1 µl of each of the forward and reverse primer stocks (10 mM), 1.5 µl of 25 mM MgCl_2_ and 0.25 µl of AmpliTaq Gold polymerase (Applied Biosystems). The following PCR conditions were applied: initial denaturation of 94°C for 10 minutes followed by 40 cycles of 94C° for 30 seconds, 58°C for 30 seconds and 72°C for 40 seconds and a final extension of 72°C for 15 minutes.

### Quantitative PCR

Quantitative real-time polymerase chain reaction was carried out on the Corbett Rotor-Gene 3000 (Corbett Research, Australia) with gene specific primers (reference genes: *BMCC1-1*, *PCA3-4* (with Exon 1/3R primer) and *β_2_M*, using Qiagen SYBR-GREEN qPCR Mastermix (QIAGEN, Germany). Each reaction contained 7.5 µl of qPCR mastermix, 5 pM of each forward and reverse primer and 5 µl of the diluted cDNA template. The following cycling conditions were applied: 95°C for 15 minutes, followed by 45 cycles of 95°C for each 20 second period, 58–59°C for 20 seconds and 72°C for 20 seconds. Data for each cycle was acquired at the elongation step and each reaction was carried out in triplicate. Relative gene expression levels were calculated using methodology described in Pfaffl [42].

## Supporting Information

Figure S1 5′RACE extension of *PCA3* mRNA from prostate cancer tissue revealed four novel transcription start sites (isoforms 1–4) located 1150 bp, 699 bp, 640 bp and 136 bp respectively upstream of the original *PCA3* start site (renamed here isoform 5). After 5′ RACE the reactions were electrophoresed on separate agarose gels beside a 100 bp size standard ladder.
(0.14 MB TIF)

Figure S2 (A) Alignment of the promoter immediately upstream of the *PCA3* isoform 4 (*PCA3-4*) transcription start site (arrowed) for different primates. Three overlapping SRY transcription factor consensus binding sites unique to the human promoter (AAACAAA - underlined) are located within the FP2 transcription factor binding footprint described by Schalken et al. [16]. (A) Alignment of the promoter immediately upstream of the PCA3 isoform 4 (*PCA3-4*) transcription start site (arrowed) for different primates. Three overlapping *SRY* transcription factor consensus binding sites unique to the human promoter (AAACAAA - underlined) are located within the FP2 transcription factor binding footprint described by Schalken et al. [16]. In the region upstream (1200 bp) of *PCA3* a similar level of sequence conservation is maintained (>85%) for the four primates (Fig 3C). However, in primates, a notable difference is observed within a transcription factor (FP2)-binding site located 195 bp upstream of the *PCA3* transcription unit previously defined using DNAse footprinting [16]. The apparent expansion of a tetranucleotide repeat ‘CAAA’ within this FP2 site in human gives rise to three overlapping consensus binding sites for SRY, a Y-linked transcription factor, that are absent from the other primates.
(0.05 MB TIF)

Figure S3 (0.17 MB TIF)

Figure S4 (0.18 MB TIF)

Table S1 BPH 3* had prostate cancer and had a TURP for bladder outflow of obstruction. All of the resected hyperplastic transition zone was scrutinised histologically and was unequivocally BPH and not PCa BPH 5 had an enucleative (Millin's-type) prostatectomy for BPH causing urinary retention: a pre-operative PSA was not performed as the patient had an indwelling urethral catheter PCa 2 & PCa 6 had previously undergone bilateral orchidectomy and had been given LHRH analogue therapy continuously, respectively, and therefore had castrate resistant prostate cancer PCa 4 commenced non-surgical androgen suppression therapy while he was recuperating from this TURP PCA 7 had a bilateral orchidectomy immediately following his TURP under the same anaesthetic an enucleative (Millin's-type) prostatectomy for BPH causing urinary retention: a pre-operative PSA was not performed as the patient had an indwelling urethral catheter PCa 2 & PCa 6 had previously undergone bilateral orchidectomy and had been given LHRH analogue therapy continuously, respectively, and therefore had castrate resistant prostate cancer PCa 4 commenced non-surgical androgen suppression therapy while he was recuperating from this TURP PCA 7 had a bilateral orchidectomy immediately following his TURP under the same anaesthetic
(0.01 MB DOC)

Table S2 The identity was based on the VISTA global alignments using human sequence as the reference. The identity was calculated by the number of identical nucleotides in an alignment divided by the length of human exon sequence. Identities <50% are not shown in the table. ^a^Nucleotide “Ns” and large gaps were excluded, so the identities in the table might be different when the whole sequences were used.
(0.01 MB DOC)
