# Supplementary figures and images for: New Genomic Structure for Prostate Cancer Specific Gene PCA3 within BMCC1: Implications for Prostate Cancer Detection and Progression
Source: PLoS One. 2009 Mar 25;4(3):e4995. doi: 10.1371/journal.pone.0004995 (PMC2655648; doi:10.1371/journal.pone.0004995)

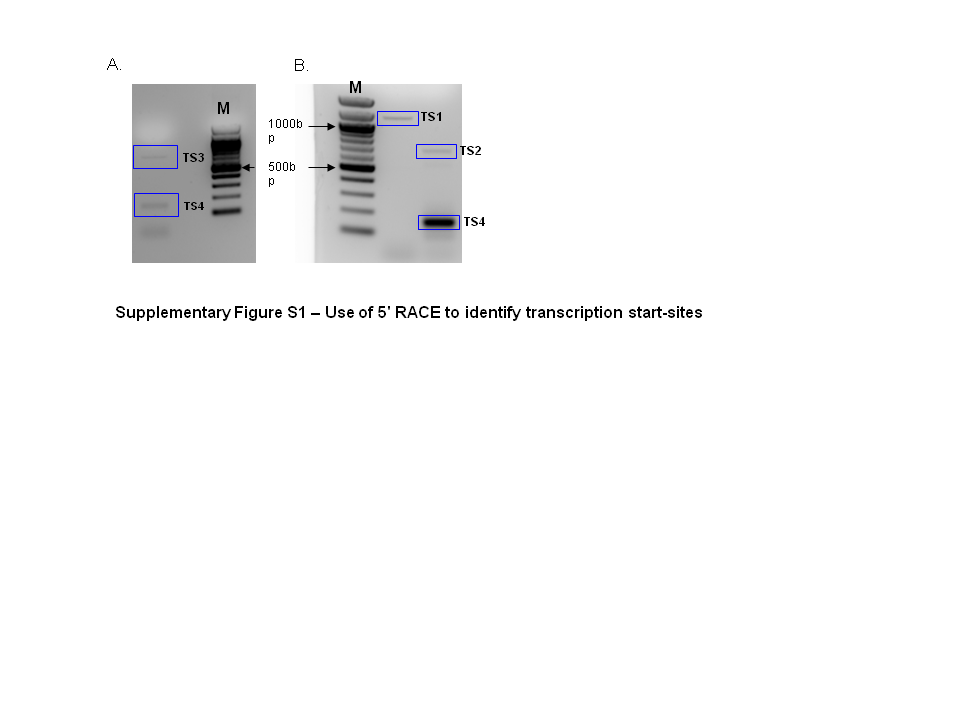

Supplement: Figure S1 — 5′RACE extension of PCA3 mRNA from prostate cancer tissue revealed four novel transcription start sites (isoforms 1–4) located 1150 bp, 699 bp, 640 bp and 136 bp respectively upstream of the original PCA3 start site (renamed here isoform 5). After 5′ RACE the reactions were electrophoresed on separate agarose gels beside a 100 bp size standard ladder. (0.14 MB TIF) [file pone.0004995.s001.tif]

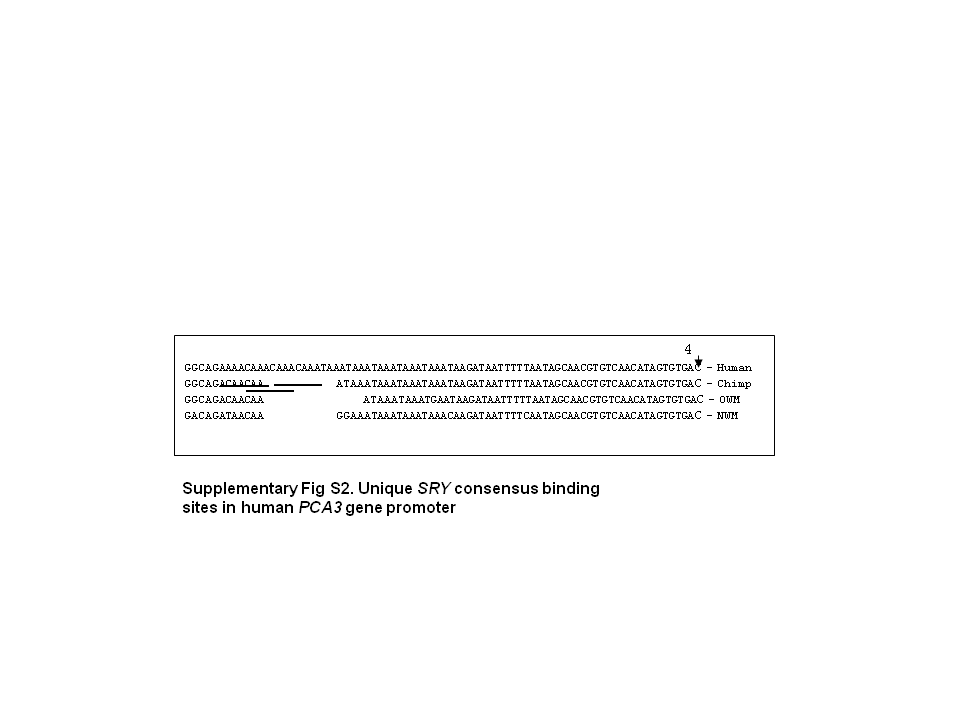

Supplement: Figure S2 — (A) Alignment of the promoter immediately upstream of the PCA3 isoform 4 (PCA3-4) transcription start site (arrowed) for different primates. Three overlapping SRY transcription factor consensus binding sites unique to the human promoter (AAACAAA - underlined) are located within the FP2 transcription factor binding footprint described by Schalken et al. [16]. (A) Alignment of the promoter immediately upstream of the PCA3 isoform 4 (PCA3-4) transcription start site (arrowed) for different primates. Three overlapping SRY transcription factor consensus binding sites unique to the human promoter (AAACAAA - underlined) are located within the FP2 transcription factor binding footprint described by Schalken et al. [16]. In the region upstream (1200 bp) of PCA3 a similar level of sequence conservation is maintained (>85%) for the four primates (Fig 3C). However, in primates, a notable difference is observed within a transcription factor (FP2)-binding site located 195 bp upstream of the PCA3 transcription unit previously defined using DNAse footprinting [16]. The apparent expansion of a tetranucleotide repeat ‘CAAA’ within this FP2 site in human gives rise to three overlapping consensus binding sites for SRY, a Y-linked transcription factor, that are absent from the other primates. (0.05 MB TIF) [file pone.0004995.s002.tif]

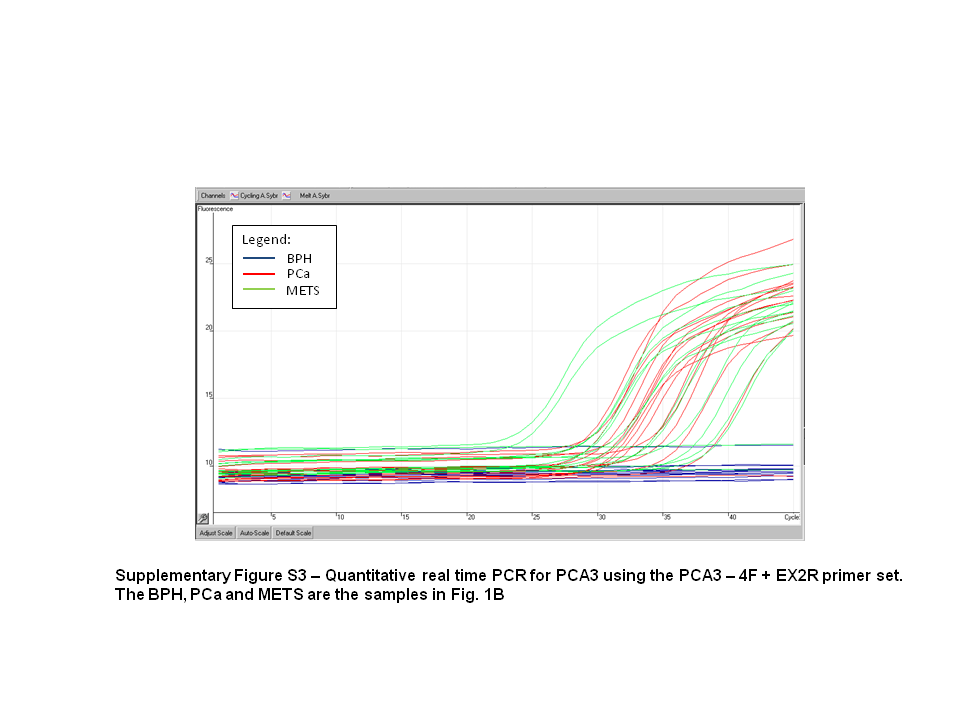

Supplement: Figure S3 — (0.17 MB TIF) [file pone.0004995.s003.tif]

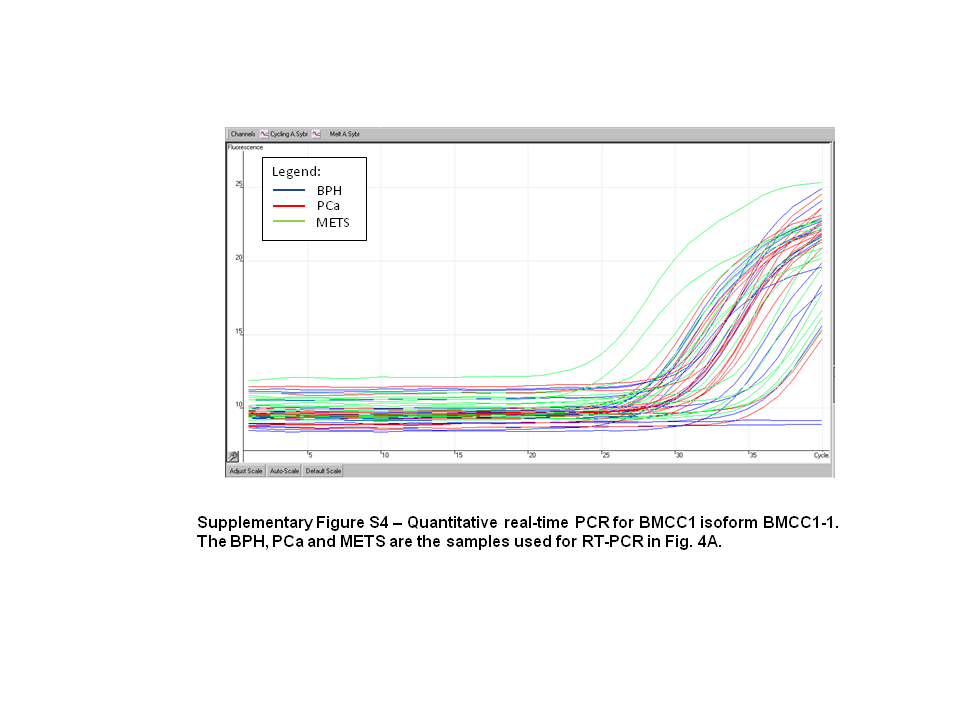

Supplement: Figure S4 — (0.18 MB TIF) [file pone.0004995.s004.tif]
