# Supplementary material for: New Genomic Structure for Prostate Cancer Specific Gene PCA3 within BMCC1: Implications for Prostate Cancer Detection and Progression
Source: PLoS One. 2009 Mar 25;4(3):e4995. doi: 10.1371/journal.pone.0004995 (PMC2655648; doi:10.1371/journal.pone.0004995)
Supplement: Table S1 — BPH 3* had prostate cancer and had a TURP for bladder outflow of obstruction. All of the resected hyperplastic transition zone was scrutinised histologically and was unequivocally BPH and not PCa BPH 5 had an enucleative (Millin's-type) prostatectomy for BPH causing urinary retention: a pre-operative PSA was not performed as the patient had an indwelling urethral catheter PCa 2 & PCa 6 had previously undergone bilateral orchidectomy and had been given LHRH analogue therapy continuously, respectively, and therefore had castrate resistant prostate cancer PCa 4 commenced non-surgical androgen suppression therapy while he was recuperating from this TURP PCA 7 had a bilateral orchidectomy immediately following his TURP under the same anaesthetic an enucleative (Millin's-type) prostatectomy for BPH causing urinary retention: a pre-operative PSA was not performed as the patient had an indwelling urethral catheter PCa 2 & PCa 6 had previously undergone bilateral orchidectomy and had been given LHRH analogue therapy continuously, respectively, and therefore had castrate resistant prostate cancer PCa 4 commenced non-surgical androgen suppression therapy while he was recuperating from this TURP PCA 7 had a bilateral orchidectomy immediately following his TURP under the same anaesthetic (0.01 MB DOC) [file pone.0004995.s005.doc]

**Supplementary Table S1 - Clinical Data**

| **Sample ID** | **Method of specimen procurement** | **Androgen Deprivation Therapy** | **Pre-procedural Serum PSA** | **Histology of Specimen** |
| --- | --- | --- | --- | --- |
| BPH 1 | TURP | Nil | 22 ng/ml | BPH |
| BPH 2 | TURP | Nil | 1.9 ng/ml | BPH |
| BPH 3 | TURP | Nil | 90 ng/ml* | BPH |
| BPH 4 | TURP | Nil | 5.7 ng/ml | BPH |
| BPH 5 | Open enucleative Prostatectomy | Nil |  | BPH |
| BPH 6 | TURP | Nil | 5.7 ng/ml | BPH |
| BPH 7 | TURP | Nil | 0.3 ng/ml | BPH |
| BPH 8 | TURP | Nil | 7.7 ng/ml | BPH |
| PCa 1 | RRP | Nil | 13.01 ng/ml | 3+4 |
| PCa 2 | TURP | Bilat orchidectomy | 56.6 ng/ml | 5+5 |
| PCa 3 | RRP | Nil | 10 ng/ml | 4+3 |
| PCa 4 | TURP | Nil | 156 ng/ml | 4+5 |
| PCa 5 | TURP | Nil | 15 ng/ml | 4+3 |
| PCa 6 | TURP | LHRH agonist | 490 ng/ml | 5+4 |
| PCa 7 | TURP | Nil | 134 ng/ml | 4+5 |
| PCa 8 | RRP | Nil | 16.3 ng/ml | 3+4 |
| Met 1 | Lap L Nodes Removed | LHRH agonist | 290 ng/ml | Metastatic PCa in lymph nodes |
| Met 2 | Lap L Nodes Removed | LHRH agonist | 90 ng/ml | Metastatic PCa in lymph nodes |
| Met 3 | Open pelvic L Node Removal | LHRH agonist | 121 ng/ml | Metastatic PCa in lymph nodes |
| Met 4 | Open pelvic L Node Removal | LHRH agonist | 64 ng/ml | Metastatic PCa in lymph nodes |
| Met 5 | Open: pelvic L Nodes | LHRH agonist | 150 ng/ml | Metastatic PCa in lymph nodes |
| Met 6 | Lap L Nodes Removed | LHRH agonist | 56 ng/ml | Metastatic PCa in lymph nodes |
| Met 7 | Lap L Nodes Removed | LHRH agonist | 172 ng/ml | Metastatic PCa in lymph nodes |
| Met 8 | Open pelvic L Node Removal | LHRH agonist | 4.5 ng/ml | Metastatic PCa in lymph nodes |
