# Supplementary material for: New Genomic Structure for Prostate Cancer Specific Gene PCA3 within BMCC1: Implications for Prostate Cancer Detection and Progression
Source: PLoS One. 2009 Mar 25;4(3):e4995. doi: 10.1371/journal.pone.0004995 (PMC2655648; doi:10.1371/journal.pone.0004995)
Supplement: Table S2 — The identity was based on the VISTA global alignments using human sequence as the reference. The identity was calculated by the number of identical nucleotides in an alignment divided by the length of human exon sequence. Identities <50% are not shown in the table. aNucleotide “Ns” and large gaps were excluded, so the identities in the table might be different when the whole sequences were used. (0.01 MB DOC) [file pone.0004995.s006.doc]

**Supplementary Table S2** Sequence identity (%) of *PCA3* exons between human and other species

| Taxonomy  (order) | Common name | Exon1  upstream  (1150 bp) | Exon 1 (120 bp) | Exon 2 (165 bp) | Exon 3 (183 bp) | Exon 4a (537 bp) | Exon 4b (1061 bp) | Exon 4c_most  conserved  part (403 bp) | Exon 4c (1856 bp) | Whole exon 4 (3455 bp) |
| --- | --- | --- | --- | --- | --- | --- | --- | --- | --- | --- |
| *Primates* | Chimpanzee | 98 | 93 | 98 | 99 | 98 | 99 | 99 | 98 | 98 |
|  | Orangutan | 94 | 97 | 94 | 98 | 97 | 95 | 98 | 96 | 96 |
|  | Rhesus macaque | 91 | 94 | 88 | 96 | 94 | 93 | 98 | 94 | 94 |
|  | Marmost | 87 | 88 | 84 | 90 | 88 | 87 | 95 | 89 | 88 |
| *Perissodactyla* | Horse |  | 68 | 77 | 72 | 78 | 77 | 95 | 72 | 75 |
| *Cetartiodactyla* | Pig | 63 | 65 | 70 | 68 | 74 | 71^a^ | 94 | 68 | 70^a^ |
| *Carnivora* | Dog | 63 | 63 | 68 | 69 | 73 | 74 | 95 | 69 | 72 |
| *Cetartiodactyla* | Cow |  |  | 66 | 73 | 70 | 74 | 94 | 67 | 70 |
| *Proboscidea* | Elephant |  |  |  | 69 | 71 | 66 | 93 | 68 | 68 |
| *Scandentia* | Treeshrew |  |  |  | 60 | 70^a^ | 67 | 92 | 59 | 63^a^ |
| *Lagomorpha* | Rabbit |  |  |  |  |  | 66 | 92 | 82^a^ | 68^a^ |
| *Rodentia* | Mouse |  |  |  |  |  | 61 | 87 | 59 | 52 |
|  | Rat |  |  |  |  |  | 59 | 86 | 58 | 51 |
| *Didelphidae* | Opossum |  |  |  |  |  |  | 91 |  |  |
